# Heparin-induced thrombocytopenia

**DOI:** 10.4103/0974-2700.76843

**Published:** 2011

**Authors:** Nissar Shaikh

**Affiliations:** Department of Anaesthesia, ICU and Pain Management, Hamad Medical Corporation, Doha, Qatar

**Keywords:** Direct thrombin inhibitors, heparin, heparin-induced thrombocytopenia, thrombosis

## Abstract

In the last 7 decades heparin has remained the most commonly used anticoagulant. Its use is increasing, mainly due to the increase in the number of vascular interventions and aging population. The most feared complication of heparin use is heparin-induced thrombocytopenia (HIT). HIT is a clinicopathologic hypercoagulable, procoagulant prothrombotic condition in patients on heparin therapy, and decrease in platelet count by 50% or to less than 100,000, from 5 to 14 days of therapy. This prothrombotic hypercoagulable state in HIT patient is due to the combined effect of various factors, such as platelet activation, mainly the formation of PF4/heparin/IgG complex, stimulation of the intrinsic factor, and loss of anticoagulant effect of heparin. Diagnosis of HIT is done by clinical condition, heparin use, and timing of thrombocytopenia, and it is confirmed by either serotonin release assay or ELISA assay. Complications of HIT are venous/arterial thrombosis, skin gangrene, and acute platelet activation syndrome. Stopping heparin is the basic initial treatment, and Direct Thrombin Inhibitors (DTI) are medication of choice in these patients. A few routine but essential procedures performed by using heparin are hemodialysis, Percutaneous Coronary Intervention, and Cardiopulmonary Bypass; but it cannot be used if a patient develops HIT. HIT patients with unstable angina, thromboembolism, or indwelling devices, such as valve replacement or intraaortic balloon pump, will require alternative anticoagulation therapy. HIT can be prevented significantly by keeping heparin therapy shorter, avoiding bovine heparin, using low-molecular weight heparin, and stopping heparin use for flush and heparin lock.

## INTRODUCTION

After 7 decades of clinical use, heparin still remains the most commonly used anticoagulant in clinical practice. Heparin use in medical practice is increasing due to the increase in the number of vascular interventions and aging population. It is estimated that up to 30% of in-hospital patients need some form of heparin during their hospital stay and 600,000 new cases of heparin-induced thrombocytopenia (HIT) are reported every year.[1]

Thrombocytopenia is one of the common abnormalities in critically ill patients. It is caused by different conditions from sepsis to intravascular devices and it is closely related to the outcome of these intensive therapy unit patients.[2]

HIT is a clinicopathologic, procoagulant condition with thrombocytopenia in patients on heparin therapy, decrease in platelet count by 50% or to less than 100 × 10^3^ /L, from 5 to 14 days of therapy.[3] HIT is associated with high morbidity, mortality, and longer hospital stay of the suffering patients.[3]

## EPIDEMIOLOGY

The incidence of HIT varies with type and duration of heparin used. Incidence is significantly lower, when low–molecular weight heparin (LMWH) is used.[4] When compared between bovine and porcine heparin, Incidence is significantly low with porcine heparin.[5] HIT is more common in surgical patients compared with medical patients, highest incidence being seen in orthopedic patients (3%–5%)[4,5] than in cardiac patients (1%–3%)[6] and it is lowest (<1%) in obstetric patients.[7]

## CLASSIFICATION

HIT is divided into 2 types, depending on whether immunologically mediated or not.

Type 1 HIT or nonimmunologically mediated thrombocytopenia or heparin-associated thrombocytopenia, is associated with a larger dose of heparin and occurs earlier, caused by direct nonimmune platelet activation by heparin; it is self-limiting, asymptomatic, and without any complications.[8]

Type 2 HIT or immunologically mediated thrombocytopenia is caused by heparin-dependent antibodies. It is subdivided into (a) heparin antibodies without any thrombocytopenia; (b) isolated HIT, thrombocytopenia with antibodies; and (c) HITT or HIT thrombotic syndrome or white clot is HIT with thrombosis.[9]

## NORMAL COAGULATION CASCADE AND THROMBIN GENERATION

**Figure 1:**
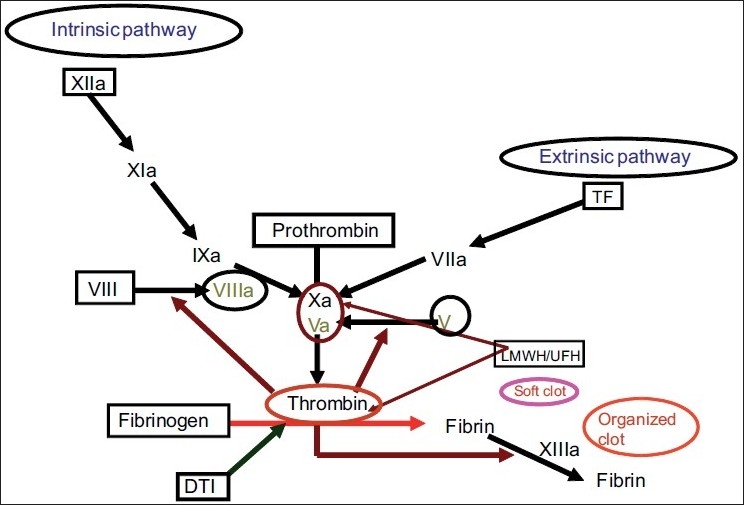
Coagulation pathway and mechanism of action of thrombin inhibitors

When due to any etiologic reason, Virchow’s triad/vascular injury occurs, the tissue factor is activated from the damaged endothelium, which combines with factor vii, and activates coagulation cascade, leading to thrombin generation. Thrombin is the chief factor in the coagulation process; it converts fibrinogen to fibrin, and also activates coagulation factors V, VIII, and XI, which in turn produce more thrombin. In combination with factor xiii, thrombin favors the formation of cross-linked hard clot Figure 1. This coagulation cascade is regulated by natural anticoagulants (tissue pathway inhibitors, antithrombin III, protein C and S); they restrict the process of clot formation to the site of injury only.[10]

## PATHOPHYSIOLOGY

Hypercoagulable state in HIT patients is multifactorial. In these patients, there is platelet activation with the formation of procoagulant PF4/heparin/immunoglobulin complex, increase in thrombin generation, activation of intrinsic tissue factor, and neutralization of anticoagulant effect of heparin.[11]

Heparin is a negatively charged polysaccharide, and it forms an immunologic complex by combining with the positively charged platelet factor 4 (PF4). PF4 is released from platelet storage granules during the activation of platelets. LMWH wraps around PF4 to a much lesser extent as compared with the unfractional heparin (UFH), hence the incidence of HIT is lower in patients on LMWH.[5] This binding of heparin to PF4, causes neutralization of the anticoagulant effect of heparin. Thus formed immune complex of heparin, PF4, and IgG bind to the FC receptor site of platelets, leading to strong platelet activation, activation of intrinsic tissue factor, and a boom in thrombin generation.[11] This overall process results in a potentially dangerous procoagulant hypercoagulable state, and a significant increased risk of thrombosis is seen in patients with thrombocytopenia (89%) in comparison to those with a normal platelet count (19%).[5]

## RISK FACTORS

Few of the main risk factors for the development of HIT are high dose of intravenous UFH, UFH, bovine heparin, and patients undergoing surgery.

## DIAGNOSIS

HIT is manifested by thrombocytopenia between 5 and 15 days of heparin treatment. Clinical scoring[12] to diagnose HIT is composed of 4 “T”s: (1) Thrombocytopenia; (2) Timing; (3) Thrombosis; and (4) The absence of other explanations. The scoring system is derived from the 4 “T”s [Table 1].

**Table 1:** Total score 0–3 low probability; 4–5 moderate probability, and 6–8 high probability of HIT

Probability of HIT	2	1	0
Thrombocytopenia	>50% platelet fall	30%–50%	<30%
Timing of thrombocytopenia	Day 5–10	> Day 10	< Day 4
Thrombosis	Proven new thrombus	Recurrent progressive Thrombosis	None
Other causes for Thrombocytopenia	None	Possible	Definite

HIT, HEPARIN-INDUCED THROMBOCYTOPENIA

Simple platelet monitoring is important for early diagnosis and prompt recognition of HIT. If a patient is on full dose of UFH, daily platelet count monitoring is a must, or receiving intermediate dose of UFH or platelets should be monitored every other day, but when the patient is receiving LMWH or low dose UFH, twice a week monitoring of platelet count is enough.[13] There are mainly 2 types of laboratory assays developed to detect HIT antigen and antibody activation assays. The activation assay is done by using washed platelets and serotonin release assay; the antigen assay is based on detecting antibodies against PF4/heparin bound. However, both these tests are in agreement in patients with clinical diagnosis of HIT.[14]

## DIFFERENTIAL DIAGNOSIS

HIT has to be differentiated from disseminated intravascular coagulation (DIC), heparin toxicity, and hyperresponsive thrombocytopenia. It is critical to differentiate these conditions from HIT as their management is totally different from that of HIT.

DIC is a pathologic activation of intravascular coagulation leading to thrombocytopenia and bleeding due to various diseases ranging from sepsis to trauma and obstetrical causes. In DIC the endovascular injury leads to small vascular thrombosis with consumption of coagulation factors and platelets. Thrombocytopenia of DIC can be differentiated from HIT by bleeding tendency, prolonged coagulation parameters, and elevated fibrinogen degradation products.[15]

Acute heparin toxicity is the heparin overdose, which can be life threatening and fatal. Common manifestations are bleeding from wound, mucosal membrane, and more critical intraventricular hemorrhage. It can be differentiated from HIT by bleeding, abnormally prolonged activated prothrombin time with thrombocytopenia.[16]

Hyperresponsive thrombocytopenia occurs in various inflammatory diseases, such as eczema, allergic rhinitis, and bronchial asthma. Particularly, the platelets are actively involved and consumed in acute bronchial asthma leading to bronchial hyperresponsiveness, bronchoconstriction with airway inflammation, and thrombocytopenia. It can be differentiated from HIT by the signs and symptoms of primary etiology leading to hyperactive response.[17]

## COMPLICATIONS ASSOCIATED WITH HIT

Mainly the following complications are seen in HIT patients: venous/arterial thrombosis, skin lesions, and acute platelet activation syndrome.

Venous thromboembolic complications are 4 times more common than arterial thrombosis.[18] Mainly thrombosis is in the larger vein, bilateral deep venous thrombosis, and pulmonary embolism, but rarely can cause thrombotic stroke, adrenal hemorrhagic infarction, venous limb gangrene, or cerebral venous sinus thrombosis. Arterial thrombosis can cause myocardial or brain infarction.[18]

Skin necrosis or erythematous plaques can occur at the site of heparin injections. Acute platelet activation syndrome will manifest as acute inflammatory response with fever and chills. Despite severe thrombocytopenia, hemorrhage is not a characteristic complication of HIT.[5]

## ALTERNATIVE TO HEPARIN

Alterative anticoagulants are required in HIT patients with thromboembolic phenomenon or routine essential procedures (hemodialysis) or emergencies, such as acute coronary syndrome.

The alternative anticoagulant should not generate HIT antibodies or cross-react with antiheparin–platelet factor 4 antibodies. The important aspects of selection of these agents are familiarity with the agent, its dosage, safety and efficacy, patient’s condition and hepatic and renal functions, clearance of agents, and availability of the monitoring techniques.[19]

The alternative anticoagulant agents to heparin are direct thrombin inhibitors (which directly inhibit thrombin generation) danaparoid, Fondaparinux (selective factor X inhibitors), and warfarin (vitamin K antagonist). Details of the pharmacodynamics and pharmacokinetics of these agents are discussed in the Treatment section.

### Treatment

As the highest risk for development of thrombosis is from the time of diagnosis of HIT to the starting of antithrombin agents, the management of HIT patient should be started without delay and waiting for the results of confirmatory laboratory tests.[13] The treatment of HIT is summarized by 6 “A”s[20]: (1) Avoid and stop all heparin (any form, any route, heparin flush, or heparinized catheters). (2) Also start direct thrombin inhibitors. (3) Anti-PF4/heparin antibody test for confirmation of diagnosis. (4) Avoid platelet transfusion. (5) Await platelet recovery. (6) Assess lower extremity thrombosis. In the management of HIT patients it is of vital importance to know what not to do [Table 2].

**Table 2:** Don’ts of HIT management

Drug	Reason
Warfarin	Warfarin in the absence of an anticoagulant can precipitate venous limb gangrene.
Platelet	Infusing platelets “adds fuel to the fire.”
Vena caval filter	Devastating caval, pelvic, lower leg venous thrombosis.
LMWH	LMWH cross-reacts with unfractionated heparin after HIT.

HIT, HEPARIN-INDUCED THROMBOCYTOPENIA; LMWH, LOW–MOLECULAR WEIGHT HEPARIN

Various anticoagulant therapies are investigated over the last decade for the treatment and prevention of new thrombosis in HIT patients. LMWH are contraindicated in patients with HIT due to its cross-reaction with heparin antibodies, and in acute HIT phase warfarin is contraindicated as it paradoxically worsens the thrombosis due to a drastic decrease in protein C levels.[21]

**Figure 2:**
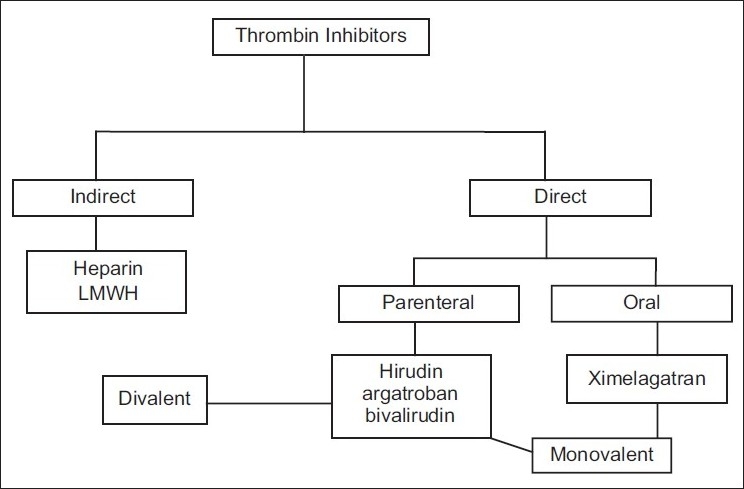
Types of thrombin inhibitors

The Direct Thrombin Inhibitors (DTI) are the medication of choice in patients with HIT or interventions where heparin is indicated but cannot be used, as these agents neither interact with heparin-dependent antibodies nor do they need an antithrombin as a cofactor [Figure 2]. DTI have a predictable anticoagulant effect. They rapidly stop the thrombin storm and some of them prevent new thrombus formation. The available DTI are argatroban, lepirudin, desirudin, bivalirudin, melagatran, and ximelagatran.[22] Depending on their structural configuration, DTI are divided into 2 groups. The lepirudin and smaller synthetic analogs (desirudin and bivalirudin) are divalent DTI, as they interact with both catalytic and substrate recognition site on thrombin. The monovalent DTI include argatroban, melagatran, and ximelagatran; these agents interact with the catalytic site of thrombin only. Group 1 DTI or divalent DTI; lepirudin and desirudin are 65-amino acid, polypeptides, the amino terminal binds to the catalytic site whereas carboxyl terminal irreversibly binds to the exosite of thrombin.[23] Bivalirudin is a 20-amino acid derivative of hirudin, the peptide bond slowly cleaved from the catalytic site on thrombin; hence it is a reversible inhibitor of thrombin with shorter half-life. Lepirudin and desirudin are given by intravenous and subcutaneous route, and half-life is 60 and 120 min, respectively. Both are excreted predominantly through the renal system; and therefore, need dose adjustment in patients with renal insufficiency. The initial loading dose is 0.4 mg/kg, then 0.15 mg/kg/h. Bivalirudin is given intravenously, largely cleared by peptidase but 20% is excreted through the kidneys, and needs dose adjustment in patients with renal impairment[23] [Table 3].

**Table 3:** Pharmacokinetics of direct thrombin inhibitors

	Argatroban	Lepirudin	Desirudin	Bivalirudin	Ximelagatran
Type of DTI	Monovalent	Divalent	Divalent	Divalent	Monovalent
Half-life	50 min	60 min	120min	36 min	1.5–4 h
Elimination	Hepatic	Renal	Renal	Dipeptidase + renal	Renal
Route of administration	Intravenous	Intravenous	Subcutaneous	Intravenous	Oral
Dosage	2 µm/kg/h	0.15 mg/kg/h	15 mg Q12 h	0.75 mg/kg/h	24 mg Q12 hourly
Monitoring	aPTT	aPTT	aPTT	ACT	No need
Risk of bleeding	Less	More	More	Less	Less

ACT, ACTIVATED CLOTTING TIME; APTT, ACTIVATED PARTIAL THROMBOPLASTIN TIME; DTI, DIRECT THROMBIN INHIBITORS

Lepirudin should be monitored 4 h after the initiation of therapy; and the target activated partial thromboplastin time (aPTT) is 1.5–2.5 times of mean reference value. Desirudin does not need monitoring except in patients with renal impairment. Bivalirudin should be monitored by activated clotting time (ACT), in patients with renal insufficiency or increased risk of bleeding. Group 2 DTI or monovalent DTI, argatroban is l-arginine-based molecule.[24] It is shorter and reversible in binding with thrombin; it has a half-life of 50 min and mainly excreted through the liver and hence needs dose adjustment in patients with hepatic insufficiency[24] [Table 3]. It is given by intravenous route and monitored with aPTT levels. Ximelagatran is a prodrug, given by the oral route and metabolized in the liver to active form, whereas melagatran has a predictable anticoagulation effect and longer half-life. Its clearance is not affected by liver impairment or moderate renal insufficiency and hence there is no need to monitor the levels unless the renal impairment is severe.[25]

Bleeding is the major concern for all DTI, risk of bleeding is higher with lepirudin and desirudin when compared with argatroban and no specific antidote is available for DTI.[24] In a prospective study using lepirudin in HIT patients, it is found that there was a significant drop in mortality, limb amputations, and new thromoembolic events in lepirudin-treated patients compared with the control group (30.9% vs 52%).[26]

In another study, Lewis *et al* demonstrated that there was a significant reduction in death, amputations, new thromboembolic events, and quick rise in platelet levels in the argatroban group compared with the control group.[27]

The warfarin therapy needed in HIT patients should be delayed till the platelet count reaches >100,000. DTI should be continued at least for 5 days with warfarin therapy and international normalizing ratio in therapeutic range for 2 days.[20,28]

A few more medications, such as danaparoid sodium and Fondaparinux, are also used in HIT patients for the treatment and prevention of thrombosis.

Danaparoid is a heparinoid compound, it interacts with antithrombin III to inhibit factor Xa. It is widely used in the treatment of HIT patients, in spite of cross-reactivity with antibodies in about 15% of the patients.[29]

Fondaparinux is a synthetic pentasaccharide that selectively inhibits factor Xa. It is FDA approved for anticoagulation and deep venous thromboprophylaxis. A small study showed no cross-reactivity of Fondaparinux with HIT antibodies.[30]

## SCENARIOS WHERE HEPARIN IS NEEDED BUT CANNOT BE USED DUE TO HIT

A few routine but essential procedures performed by using heparin are hemodialysis, Percutaneous Coronary Intervention (PCI), and Cardiopulmonary Bypass (CPB); but cannot be used if the patient develops HIT. HIT patients with unstable angina, thromboembolism, indwelling devices, such as valve replacement or intraaortic balloon pump, will require alternative anticoagulation therapy.

### Hemodialysis

HIT antibodies are positive in up to 17% of the patients on hemodialysis and mortality in these patients is significantly high. The manifestation of HIT in these patients varies from acute systemic reaction to frequent clotting in the extracorporeal circuit or increase in the number of failed arteriovenous fistula.[31]

As soon as HIT is suspected in these patients, all forms of heparin should be stopped and start DTI or danaparoid or regional citrate anticoagulation. Argatroban has advantage in these patients as no dose adjustment is required; the recommended dose is an initial bolus of 250 mcg/kg at the start of dialysis then continuous infusion of 2 mcg/kg/min until 1 h before the end of dialysis session. Only bolus dose of lepirudin recommended at the beginning of dialysis session. This DTI therapy should be monitored with aPTT and dose can be adjusted accordingly. For initial 2 dialysis sessions, the dose of danaparoid is 2500 units, then in the subsequent dialysis sessions the dose should be decreased to 2000 units and anti-Xa has to be monitored and maintained in the range of 0.5–0.8 units/mL.[32]

## PERCUTANEOUS CORONARY INTERVENTIONS AND CARDIOPULMONARY BYPASS

Hypercoagulability in HIT patients in combination with endovascular disruptions PCI and CPB will particularly increase the risk of thrombosis. Argatroban, bivalirudin, and danaparoid are commonly used in PCI and the dosages are as follows: an initial bolus dose of bivalirudin should be 0.75 mg/kg, followed by 1.75 mg/kg/min; argatroban bolus of 350 mcg/kg followed by 25 mcg/kg/min infusion with ACT monitoring; and danaparoid 2200 unit bolus followed by 150–200 units/h with anti-Xa levels monitoring.

If possible CPB surgeries should be postponed till PF4-heparin antibodies negative. If surgery has to be done, bivalirudin, lepirudin, argatroban, or danaparoid can be used.

### Unstable coronary syndrome

These patients may need full anticoagulation for days; intravenous argatroban is used successfully in HIT patients with unstable coronary syndrome.[33]

### Multiple organ failure and HIT

In patients with multiple organ dysfunction/failure who are critically ill and may have hepatic/renal impairment or failures, the dose of DTI should be adjusted with meticulous monitoring of the coagulation parameters, but drug accumulation and lack of antidote will put these critical patients at the risk of potential side effects. Bivalirudin demonstrated better safety as it is cleared predominantly by the enzymatic cleavage.

### Pregnancy and HIT

When a HIT patient becomes pregnant may require thromboprophylaxis and/or treatment for thrombosis; danaparoid, subcutaneous lepirudin, and Fondaparinux are used successfully, but limited literature is available about their effects on fetus and newborn.[34]

## MORBIDITY AND MORTALITY

Early diagnosis and management of HIT can limit the morbidity and mortality of suffering patients. Reported mortality is up to 30%, and as high as 20% of HIT patients with thrombosis need amputation.[35] In HIT patients treated with DTI, mortality decreases to 16% and the incidence of new thrombus decreases to 5.8%.[36]

Many other therapies and medications, such as antiplatelet agent and ancrod defibrogenating snake venom, are tried but showed poor efficacy and increase in thrombin generation.[37]

Small doses of thrombolytic agents were used locally in HIT patients with good results in massive pulmonary embolism or arterial thrombosis.[38]

Ralph–Edward successfully managed a case of massive pulmonary embolism in a patient with HIT by embolectomy.[39]

## PREVENTION

The following measures will decrease the incidence of HIT as well as coagulation disorders.[41]

1. Keeping heparin therapy for shorter duration and starting warfarin early if expecting prolonged anticoagulation.
2. Avoiding bovine and fractional heparin and using LMWH.[5,40]
3. Stopping the use of heparin flush for central and arterial catheters.
4. Heparin-free dialysis and not using heparin lock.

## CONCLUSION

Heparin is the commonly used anticoagulant; about 30% of hospitalized patients are exposed to some form of heparin. Most feared complication of heparin use is HIT. HIT is a clinicopathologic, procoagulant condition with thrombocytopenia in patients on heparin therapy, with decrease in platelet count by 50% or to less than 100 × 103/L, from 5 to 14 days of the therapy. It is diagnosed by 4 “T”s thrombocytopenia, timing, thrombosis absence of other causes for thrombocytopenia, and the diagnosis is confirmed by immunoassay. HIT is common in surgical and orthopedic patients. The risk of HIT is more with high dose of intravenous heparin and bovine heparin. Deep venous thrombosis and pulmonary artery embolism are common in HIT patients. Management of HIT is to stop heparin, and DTI are backbone of HIT therapy. Few cases of successful pulmonary embolectomy and arterial thrombolysis in HIT patients with massive pulmonary and arterial thrombosis are reported. Mortality in HIT patients can be as high as 30%, but it can be significantly reduced with the use of DTI to 16%. A few routine but essential procedures performed by using heparin are hemodialysis, PCI, and CPB; but cannot be used if the patient develops HIT. HIT patients with unstable angina, thromboembolism, indwelling devices, such as valve replacement or intraaortic balloon pump, will require alternative anticoagulation therapy. HIT can be prevented significantly by keeping heparin therapy shorter, avoiding bovine heparin, using LMWH, and by stopping the use of heparin flush and heparin lock.
